# Quantitative assessment of the effect of uracil-DNA glycosylase on amplicon DNA degradation and RNA amplification in reverse transcription-PCR

**DOI:** 10.1186/1743-422X-2-29

**Published:** 2005-04-11

**Authors:** Steven B Kleiboeker

**Affiliations:** 1 Veterinary Medical Diagnostic Laboratory and Department of Veterinary Pathobiology, College of Veterinary Medicine, University of Missouri, Columbia, Missouri 65211, USA

## Abstract

Although PCR and RT-PCR provided a valuable approach for detection of pathogens, the high level of sensitivity of these assays also makes them prone to false positive results. In addition to cross-contamination with true positive samples, false positive results are also possible due to "carry-over" contamination of samples with amplicon DNA generated by previous reactions. To reduce this source of false positives, amplicon generated by reactions in which dUTP was substituted for dTTP can be degraded by uracil DNA glycosylase (UNG). UNG does not degrade RNA but will cleave contaminating uracil-containing DNA while leaving thymine-containing DNA intact. The availability of heat-labile UNG makes use of this approach feasible for RT-PCR. In this study, real-time RT-PCR was used to quantify UNG degradation of amplicon DNA and the effect of UNG on RNA detection. Using the manufacturers' recommended conditions, complete degradation of DNA was not observed for samples containing 250 copies of amplicon DNA. Doubling the UNG concentration resulted in degradation of the two lowest concentrations of DNA tested, but also resulted in an increase of 1.94 cycles in the C_T _for RNA detection. To improve DNA degradation while minimizing the effect on RNA detection, a series of time, temperature and enzyme concentrations were evaluated. Optimal conditions were found to be 0.25 U UNG per 25 μl reaction with a 20 min, 30°C incubation prior to RT-PCR. Under these conditions, high concentrations of amplicon DNA could be degraded while the C_T _for RNA detection was increased by 1.2 cycles.

## Background

Molecular techniques have provided a valuable approach for detection of pathogens in both human and veterinary medicine [1-5]. As with any diagnostic technique, quality control of individual steps is critical to ensure the accuracy of results. When performing diagnostic PCR or reverse transcription (RT)-PCR, elimination of false positive results is crucial to ensuring diagnostic accuracy. False positives can occur due to contamination at any point in sample preparation and amplification procedures [6]. For example, cross-contamination between positive and negative samples may occur during sample collection, nucleic acid extraction, PCR or RT-PCR reaction assembly or during agarose gel electrophoresis analysis. In addition to contamination by positive samples, false positive results are also possible due to contamination of samples at any point in the protocol with DNA generated by previous positive amplification reactions. This source of contamination is of particular concern since a positive amplification reaction can generate in excess of 10^11 ^molecules of product (amplicon) DNA per reaction. Given that ten or fewer DNA template molecules can generate a positive result by PCR or RT-PCR, even minute levels of amplicon contamination can result in false positive results. Furthermore, the inherent stability of DNA under a variety of environmental conditions could potentially lead to false positive results weeks or months after contamination of reagents or equipment with amplicon DNA.

Uracil-DNA glycosylase (UNG) is a DNA repair enzyme that will cleave uracil-containing DNA while leaving the natural, thymine-containing DNA unaffected [7,8]. During PCR, deoxyuridine triphosphate (dUTP) can be substituted for deoxythymidine triphosphate (dTTP) in the synthesis of product DNA. Thus to reduce the frequency of false positive results due to amplicon contamination, one common recommendation [9-12] has been to substitute dUTP for dTTP as a source of nucleotides for the PCR reaction. Amplicon DNA that has incorporated dUTP can then be degraded with uracil-DNA glycosylase prior to subsequent amplification reactions, thus preventing these molecules from producing false positive results by acting as template. This approach to elimination of carry-over contamination has led several manufacturers of commercial PCR and RT-PCR reagents to substitute dUTP in the reaction mixture in place of dTTP and in the case of some manufacturers to include UNG as a standard reagent in kits. The success of this approach for elimination of contaminating amplicon DNA depends on the availability of a heat-labile UNG enzyme, the heat-inactivation of which prevents cleavage of product DNA amplified from the target template. The half-life of heat-labile UNG has been estimated to be 2 minutes at 40°C [13] thus making the use of this enzyme feasible for both PCR and RT-PCR applications since the reverse transcription step is commonly performed at 45 – 50°C for 30 min or longer.

Real-time RT-PCR utilizes fluorescence to detect the presence of amplification products as the reaction occurs. The cycle at which a positive reaction is first detectible, termed the cycle threshold (C_T_) is proportionate to the concentration of template in a sample. Real-time PCR and RT-PCR amplification products are typically much shorter (e.g. 75 – 150 bases in length) compared to those generated by standard PCR and RT-PCR assays. In addition to rapid quantification of template RNA, real-time PCR and RT-PCR offers significant advantages over standard PCR and RT-PCR for detection of DNA or RNA in terms of reduced sample handling, the time required for analysis and analytical sensitivity. However, most importantly real-time assays have reduced (though certainly not eliminated) the opportunities for false positive results due to cross-contamination of samples since real-time assays are conducted in a "closed-tube" system, in which the tubes are not opened after amplification is complete. Nonetheless, given the rigorous standards in place for both human and veterinary diagnostic laboratories and the significant consequences of false positive results, even laboratories using real-time methods may employ strategies such as UNG addition prior to RT-PCR to reduce the potential for false positive results.

While the use of UNG to eliminate amplicon contamination has been previously reported for RT-PCR assays [14,15], the effect of UNG on quantitative assay sensitivity for RNA detection has not been investigated to date. Nor has a quantitative assessment of the concentrations of contaminating DNA that can be degraded prior to RT-PCR been performed. Real-time (quantitative) RT-PCR detection of Porcine arterivirus (family Arteriviridae, order Nidovirales) RNA was used for these assessments. This virus is an important pathogen of swine and is thus frequently the target of diagnostic investigation with RT-PCR representing the principal assay for pathogen detection in many laboratories. Some high-value swine herds are free of this virus thus making the report of false positive results particularly troublesome since depopulation is a common method used to eliminate this virus from a herd. In this study, it was demonstrated that heat-labile UNG had a concentration, temperature and time-dependent effect on quantitative RT-PCR sensitivity and DNA degradation. Conditions were optimized so that minimal effects on target RNA amplification sensitivity were observed while maximizing the ability to degrade carry-over amplicon DNA contamination in a sample.

## Results

### Effect of UNG concentration on DNA degradation and RT-PCR amplification of RNA

To assess the effect of UNG on DNA degradation and RNA detection, reactions were performed under conditions recommended but the supplier of UNG (Table 1). The template for amplification was 10-fold serial dilutions of viral RNA or amplicon DNA. The amplicon DNA was from a previous RT-PCR reaction in which dUTP was used in place of dTTP. An enzyme concentration and temperature-dependent increase was observed for the C_T _of both DNA and RNA detection. However, at the enzyme concentration recommended by the supplier (0.5 U UNG per 25 μl reaction), complete degradation of amplicon DNA was not observed at 15°C – 25°C, even in reactions containing less than 250 copies (Table 1). At double the recommended enzyme concentration (1.0 U UNG per 25 μl reaction), complete degradation of amplicon DNA was observed only at 25°C in the two dilutions containing the lowest concentrations of amplicon DNA. Using UNG concentrations recommended by the supplier, increases in RNA C_T _values were noted ranging from 0.28 cycles, for 0.5 U UNG/reaction and a 15°C incubation, to 1.94 cycles for 1 U UNG/reaction and a 25°C incubation.

**Table 1:** Effect of UNG concentration and incubation temperature on DNA degradation and RNA detection^a^

		0.5 U UNG						1.0 U UNG					
			
		15°C		20°C		25°C		15°C		20°C		25°C	
							
Analyte (dil.)	Control^c^	C_T_	incr.	C_T_	incr.	C_T_	incr.	C_T_	incr.	C_T_	incr.	C_T_	incr.
RNA (undil.)^b^	21.21	21.77	0.56	21.38	0.17	21.81	0.60	22.04	0.83	21.97	0.76	23.34	2.13
RNA (1:10)	24.91	25.11	0.20	25.29	0.38	25.44	0.53	25.10	0.19	26.23	1.32	26.69	1.78
RNA (1:100)	28.32	28.05	-0.27	28.41	0.09	29.23	0.91	28.47	0.15	29.73	1.41	30.13	1.81
RNA (1:1000)	31.16	31.78	0.62	31.59	0.43	32.15	0.99	32.70	1.54	32.70	1.54	33.19	2.03
													
DNA (1:10^7^)^b^	21.20	24.22	3.02	26.04	4.84	27.54	6.34	24.67	3.47	28.09	6.89	30.45	9.25
DNA (1:10^8^)	23.91	28.01	4.10	29.20	5.29	31.46	7.55	28.06	4.15	32.30	8.39	34.98	11.07
DNA (1:10^9^)	27.09	31.71	4.62	32.89	5.80	33.50	6.41	32.00	4.91	36.02	8.93	No C_T_^e^	-
DNA (1:10^10^)	30.48	34.43	3.95	36.00	5.52	36.24	5.76	36.78	6.30	39.03	8.55	No C_T_	-
Mean C_T _increase for RNA			0.28		0.27		0.76^d^		0.68		1.26^d^		1.94^d^
Mean C_T _increase for DNA			3.92		5.36		6.52		4.71		8.19		10.16

^a ^Incubations were performed before RT-PCR with the indicated concentrations of UNG per 25 μl reaction at the indicated temperatures for 10 min.

^b ^The undiluted RNA sample and the 1:10^7 ^dilution of the DNA sample contained 250,000 copies of RNA or DNA, respectively

^c ^Control reactions did not contain UNG and were not incubated prior to RT-PCR

^d ^P < 0.05 by paired t-test compared to control values

^e ^Indicates that amplification was not detected through 40 cycles in each of three replicate reactions

To further optimize the effect of UNG on DNA degradation and minimize the effect of UNG on RNA amplification, RT-PCR reactions were performed containing a range of UNG concentrations with a longer incubation and higher temperature than recommended by the enzyme supplier (Table 2). A concentration-dependent effect was observed for DNA degradation, with the lowest UNG concentration tested (0.1 U per 25 μl reaction) increasing the C_T _for DNA detection but failing to completely eliminate DNA contamination even at the lowest concentration tested. The highest concentrations tested, 0.5 and 1.0 units of UNG per 25 ul reaction, completely eliminated all detectible uracil-containing DNA, including the highest concentration of amplicon DNA tested which contained approximately 250,000 copies in a 25 μl reaction.

**Table 2:** Effect of UNG concentration on DNA degradation and RNA detection^a^

		0.1 U UNG		0.25 U UNG		0.5 U UNG		1.0 U UNG	
					
Analyte (dil.)	Control^c^	C_T_	incr.	C_T_	incr.	C_T_	incr.	C_T_	incr.
RNA (undil.)^b^	21.26	22.30	1.04	22.79	1.53	23.09	1.83	23.86	2.60
RNA (1:10)	26.25	26.86	0.61	27.57	1.32	27.95	1.70	27.31	1.06
RNA (1:100)	31.99	32.52	0.53	33.15	1.16	33.47	1.48	34.04	2.05
RNA (1:1000)	35.08	35.25	0.17	36.73	1.65	36.86	1.78	No Ct	-
									
DNA (1:10^7^)^b^	21.53	26.56	5.03	35.04	13.51	No Ct	-	No Ct	-
DNA (1:10^8^)	24.53	30.24	5.71	38.34	13.81	No Ct	-	No Ct	-
DNA (1:10^9^)	28.01	33.38	5.37	No Ct^e^	-	No Ct	-	No Ct	-
DNA (1:10^10^)	30.82	36.44	5.62	No Ct	-	No Ct	-	No Ct	-
Mean C_T _increase for RNA			0.59^d^		1.42^d^		1.70^d^		1.90^d^
Mean C_T _increase for DNA			5.43		13.66		N/A		N/A

^a ^Incubation was performed before RT-PCR at 30°C for 30 min at the indicated enzyme concentrations

^b ^The undiluted RNA sample and the 1:10^7 ^dilution of the DNA sample contained 250,000 copies of RNA or DNA, respectively

^c ^Control reactions did not contain UNG and were not incubated prior to RT-PCR

^d ^P < 0.05 by paired t-test compared to control values

^e ^Indicates that amplification was not detected through 40 cycles in each of three replicate reactions

An UNG concentration-dependent effect was also observed for RNA detection by RT-PCR (Table 2). The lowest UNG concentration tested, 0.1 U per 25 μl reaction, increased the mean C_T _of RNA detection by 0.59 cycles, while the highest concentration of UNG tested, 1.0 U per 25 μl reaction, increased the mean C_T _of RNA detection by 1.90 cycles. Intermediate C_T _increases were observed with 0.25 and 0.5 U UNG per reaction. The increases in C_T _values for RNA detection following incubation with UNG correspond to 1.5 – 3.7-fold decreases in detectible RNA.

### Effect of incubation temperature and time on DNA degradation and RT-PCR amplification of RNA

The effect of temperature on DNA degradation and RNA amplification in the presence of UNG was assessed at three incubation temperatures prior to RT-PCR (Table 3). Incubation at 25°C prior to RT-PCR increased the C_T _for DNA detection by 8.67 cycles but did not completely eliminate a positive reaction even at the lowest concentration of amplicon DNA. Incubation at 30°C and 35°C eliminated positive reactions at the three lowest concentrations of DNA and increased the C_T _for the highest DNA concentration by 16.03 and 17.09 cycles, respectively. A temperature dependent increase in C_T _for RNA amplification was also noted, with incubation temperatures of 25°C and 30°C resulting in smaller increases in C_T _values for RNA detection than incubation at 35°C.

**Table 3:** Effect of incubation temperature on DNA degradation and RNA detection in the presence of UNG^a^

		25°C		30°C		35°C	
				
Analyte (dil.)	Control^c^	C_T_	incr.	C_T_	incr.	C_T_	incr.
RNA (undil.)^b^	21.22	22.40	1.18	23.17	1.95	23.79	2.57
RNA (1:10)	26.15	26.93	0.78	27.54	1.39	28.25	2.10
RNA (1:100)	31.37	32.51	1.14	32.05	0.68	33.79	2.42
RNA (1:1000)	33.62	35.46	1.84	35.24	1.62	37.43	3.81
							
DNA (1:10^7^)^b^	21.48	32.11	10.63	37.51	16.03	38.57	17.09
DNA (1:10^8^)	24.89	34.33	9.44	No Ct^e^	-	No Ct	-
DNA (1:10^9^)	28.22	36.08	7.86	No Ct	-	No Ct	-
DNA (1:10^10^)	31.75	38.48	6.73	No Ct	-	No Ct	-
Mean C_T _increase for RNA			1.24^d^		1.41^d^		2.73^d^
Mean C_T _increase for DNA			8.67		16.03		17.09

^a ^The UNG concentration was 0.25 units per 25 μl reaction and incubation time before RT-PCR was 30 min at the indicated temperatures.

^b ^The undiluted RNA sample and the 1:10^7 ^dilution of the DNA sample contained 250,000 copies of RNA or DNA, respectively

^c ^Control reactions did not contain UNG and were not incubated prior to RT-PCR

^d ^P < 0.05 by paired t-test compared to control values

^e ^Indicates that amplification was not detected through 40 cycles in each of three replicate reactions

Incubation times with UNG of 10, 20, and 30 minutes were evaluated for DNA degradation and the effect on RNA amplification by RT-PCR (Table 4). An incubation of 10 min eliminated DNA detection at the lowest concentration and increased the C_T _by a mean of 6.58 cycles for the other DNA dilutions. Incubation times of 20 and 30 min eliminated progressively more DNA from the reactions. A time-dependent increase in C_T _values was also observed for RNA detection by RT-PCR, with a 30 min incubation with UNG resulting in the greatest mean increase of 1.42 cycles in the C_T_.

**Table 4:** Effect of incubation time on DNA degradation and RNA detection in the presence of UNG^a^

		10 min		20 min		30 min	
				
Analyte (dil.)	Control^c^	C_T_	incr.	C_T_	incr.	C_T_	incr.
RNA (undil.)^b^	21.07	21.79	0.72	22.69	1.62	22.74	1.67
RNA (1:10)	25.80	25.96	0.16	26.83	1.03	27.51	1.71
RNA (1:100)	31.28	31.74	0.46	32.15	0.87	32.78	1.50
RNA (1:1000)	34.81	36.16	1.35	36.01	1.20	35.6	0.79
							
DNA (1:10^7^)^b^	21.46	28.19	6.73	32.71	11.25	35.93	14.47
DNA (1:10^8^)	24.73	31.22	6.49	36.52	11.79	No Ct	-
DNA (1:10^9^)	28.08	34.60	6.52	No Ct	-	No Ct	-
DNA (1:10^10^)	31.14	No Ct^e^	-	No Ct	-	No Ct	-
Mean C_T _increase for RNA			0.67		1.18^d^		1.42^d^
Mean C_T _increase for DNA			6.58		11.52		14.47

^a ^The UNG concentration was 0.25 units per 25 μl reaction and incubation before RT-PCR was performed at 30°C for the indicated times.

^b ^The undiluted RNA sample and the 1:10^7 ^dilution of the DNA sample contained 250,000 copies of RNA or DNA, respectively

^c ^Control reactions did not contain UNG and were not incubated prior to RT-PCR

^d ^P < 0.05 by paired t-test compared to control values

^e ^Indicates that amplification was not detected through 40 cycles in each of three replicate reactions

### Simultaneous detection of RNA amplification and DNA degradation

**Figure 1:**
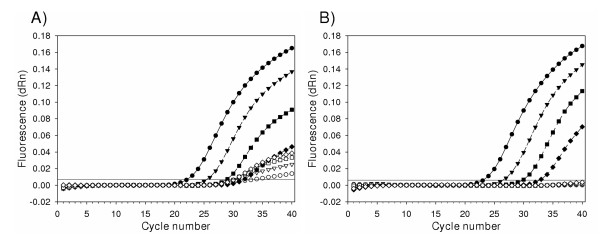
Simultaneous detection of viral RNA and contaminating DNA. Serial ten-fold dilutions of viral RNA with approximately 1,000 copies of heterologous competitor (amplicon) DNA per reaction were amplified following incubation for 20 min at 30°C either without (A) or with (B) 0.25 U UNG per reaction. Reactions were performed in triplicate and contained approximately 250,000 copies viral RNA (●), 25,000 copies viral RNA (▼), 2,500 copies viral RNA (■) or 250 copies viral RNA (◆). Each reaction contained 1,000 copies of amplicon DNA. The fluorescence signal generated by amplicon DNA is indicated by the open form of the same symbol for each respective reaction. The horizontal line at approximately 0.008 fluorescence units (dRn) indicates the threshold for a positive reaction. dRn, baseline-corrected normalized fluorescence.

To ensure that significant levels of contaminating DNA could be degraded at the same time that RNA was amplified and quantified by real-time RT-PCR, viral RNA was contaminated with amplicon DNA prior to UNG incubation and RT-PCR amplification (Fig. 1). To accomplish this, a constant amount of amplicon DNA generated from a heterologous competitor (which has the viral oligonucleotide primer binding sites but a different recognition sequence for the dual-labeled TaqMan oligonucleotide probe) was added to ten-fold serial dilutions of viral RNA. In reactions which did not contain UNG, approximately 1,000 copies of contaminating DNA were detected in each dilution of viral RNA (Fig. 1A). However, addition of 0.25 U UNG per reaction with incubation at 30°C for 20 min prior to RT-PCR completely eliminated the signal for contaminating DNA in each reaction (Fig. 1B). The amplification curves for viral RNA detection were unaffected other than an increase in C_T _by a mean of 1.2 cycles, a value consistent with results shown above.

### Effect of UNG and incubation prior to RT-PCR on standard curves for RNA quantification

**Figure 2:**
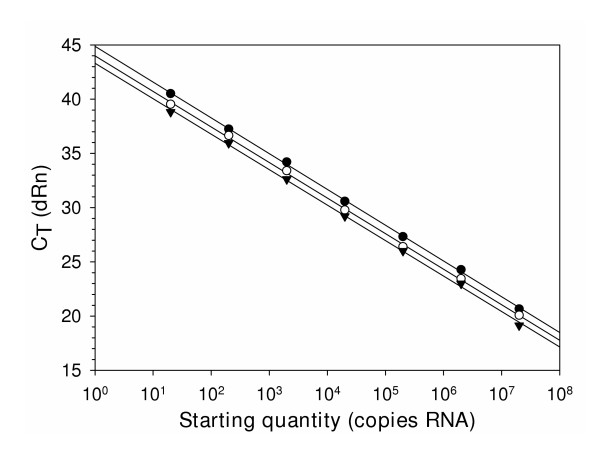
Effect of UNG on Standard curves for competitor RNA. Linear regression analysis for each standard curve was performed within the analysis software (Stratagene Mx4000 version 4.00). Symbols represent means for samples analyzed in triplicate. Addition of 0.25 units UNG per reaction followed by incubation at 30°C for 20 min prior to RT-PCR (●), no addition of UNG but incubation at 30°C for 20 min prior to RT-PCR (○), no addition of UNG and no incubation prior to RT-PCR (▼). C_T_, cycle threshold. dRn, baseline-corrected normalized fluorescence.

To assess the effect of UNG addition to reaction mixtures on standard curves used for RNA quantification, reactions were performed using 10-fold dilutions of heterologous competitor RNA (Fig. 2). Standard curves were analyzed for reactions without UNG or containing 0.25 units UNG and were incubated 30 min at 30°C prior to RT-PCR. Regression analyses for these curves were compared to a standard curve using the same RNA dilutions which were not incubated at 30°C for 20 min prior to RT-PCR. Regression analysis for all three reaction conditions demonstrated parallel lines with R^2 ^values >0.99. The slopes of the regression lines were essentially equal at approximately -3.330, which indicate amplification efficiencies near 100%. Only the y-intercepts differed between the three reaction conditions, with the values from the reactions containing UNG and incubated at 30°C for 20 min slightly greater than the standards that did not contain UNG and were not incubated prior to RT-PCR. The y-intercept value for the reactions not containing UNG and incubated at 30°C for 20 min prior to RT-PCR was intermediate of the other two values.

### Detection of low viral RNA concentrations in reactions containing UNG

To determine if UNG addition and incubation prior to RT-PCR would result in false negative results under the conditions described, 96 replicate samples containing approximately 20 copies of viral RNA per replicate were amplified by RT-PCR. Of the 96 replicate samples tested, positive amplification (defined as C_T _< 40) was not detected in three samples that contained 0.25 units UNG and were incubated at 30°C for 30 min prior to RT-PCR (data not shown). Of 96 control reactions (i.e. no UNG added and no incubation prior to RT-PCR), amplification was detected in all but one of the replicates. The mean C_T _increase of samples containing UNG and incubated at 30°C for 30 min prior to RT-PCR was 1.56 cycles, a value in agreement with results shown above.

## Discussion

The techniques of PCR and RT-PCR offer several advantages when compared to traditional viral diagnostic techniques, especially in terms of analytical sensitivity and time for assay completion. Unfortunately the high level of sensitivity, which approaches the single molecule level, also makes this technique prone to false positive results. Amplicon generated by previous positives reactions in which dUTP was substituted for dTTP can be degraded by UNG and thus theoretically eliminated as a source of template that would cause false positives in PCR or RT-PCR. UNG degrades contaminating uracil-containing DNA while leaving the natural, thymine-containing DNA intact. The precise, reproducible quantification of real-time RT-PCR provides a rapid method to assess and optimize the use of UNG to eliminate or reduce the impact of amplicon DNA in RT-PCR as well as determine the effects of UNG on RNA detection. The manufacturers' recommendation for the use of heat-labile UNG include addition of 1 unit enzyme per 50 μl reaction followed by an incubation time of 10 min at 15 – 25°C. In this study, this enzyme concentration and the incubation conditions were not found to eliminate amplicon DNA, presumably due at least in part to the short length of amplicon DNA (114 bases). To consistently degrade approximately 500 copies of amplicon DNA, a two-fold increase in UNG concentration was necessary. However, this concentration of UNG resulted in a nearly two cycle increase to reach the threshold for RNA detection. These results are corroborated by a recent publication using real-time PCR for detection of single-copy genes in which it was concluded that manufacturers' recommended conditions were not adequate to consistently degrade even minimal (e.g. < 30 copies) amounts of amplicon DNA [16].

To determine optimal conditions for amplicon degradation with minimal effect on RNA detection by RT-PCR, a series of time, temperature and enzyme concentrations were evaluated. From these results, it was shown that UNG degradation of amplicon DNA in RT-PCR was concentration, time and temperature depended, as previously described for real-time PCR [16]. To allow degradation of considerable concentrations of carry-over amplicon DNA while having minimal effects on RNA detection, the optimal concentration of UNG was found to be 0.25 U per 25 μl reaction with a pre-amplification incubation at 30°C for 20 min. This incubation was performed after the complete reaction had been assembled in the reaction tubes, just prior to the RT step. These conditions completely degraded approximately 2,500 copies of carry-over amplicon DNA while reducing the detectible concentration of viral RNA template by 2.2-fold (i.e. C_T _values increased by a mean of 1.2 cycles). If higher levels of carry-over contamination are encountered, increasing the incubation time by an additional 10 min demonstrated degradation of a 10-fold higher concentration of amplicon DNA while only slightly increasing the C_T _for RNA detection. It is interesting to note that, based on analysis of the standard curves, increases observed in the C_T _for RNA were due both to the presence of UNG and the incubation at 30°C prior to RT-PCR.

Given the relatively long time required for the reverse transcriptase step, a heat-labile UNG that is rapidly and effectively inactivated at temperatures below that of the RT step must be used for this approach to be applied to control false positive reactions in RT-PCR. The commercially available heat-labile UNG used in this study is rapidly inactivated at 40°C with a half-life of 2 min [13]. At the end of the UNG incubation, the samples were held at 55°C to rapidly inactivate UNG just prior to the RT step which was 50°C for 30 min. For the RT-PCR reagents used (as well as many other commercially availably RT-PCR reagents), the manufacture states that the 30 min RT step can be performed at temperatures of up to 55°C (or higher for some reagent systems), and reactions analyzed in preliminary experiments (data not shown) demonstrated no effect on RT-PCR analytical sensitivity following a 2 min incubation at 55°C prior to the RT step.

Results presented herein demonstrated that incubation with UNG appears to increase the C_T _equally for in vitro transcribed RNA and viral RNA, thus quantification through the use of a standard curve can remain accurate provided that all reactions are performed under the same conditions. However, a small percentage of weak positive samples may not be detected due to an increase in C_T _caused by UNG and incubation prior to RT-PCR. To the reduce the possibility of false negatives with dilute RNA samples, results from this study suggest that replicates of two or more reactions should be sufficient, given that the false negative rate was only increased by 2% for samples containing UNG and incubated prior to RT-PCR. Also, two – five additional cycles of amplification may provide positive amplification of target RNA in samples with very low concentrations.

## Conclusion

Quantitative assessment of the effect of UNG on DNA degradation and RNA amplification over a range of enzyme concentrations, temperatures and times demonstrated that optimization of reaction conditions allows selection of conditions that maximize carry-over amplicon degradation while minimizing the effect on RNA detection. While this study was performed with real-time RT-PCR to provide accurate quantification of the effects of UNG, these findings are potentially useful to both standard and real-time RT-PCR amplification methods.

## Methods

### Quantitative (TaqMan) RT-PCR

Amplification reactions were performed using the Qiagen QuantiTect Probe RT-PCR kit (Qiagen, Inc., Valencia, CA) with thermocycling and detection performed in a Stratagene Mx4000 real-time PCR machine (Stratagene, Inc., La Jolla, CA). Samples were analyzed in triplicate. The amplification protocol, oligonucleotide primers and dual-labeled probe used for 5' exonuclease (TaqMan) amplification of the North American PRRSV Ingelvac MLV were as previously described [17] and amplified a 114-bp fragment. The amplified in the presence of dUTP, the sense strand and anti-sense strand will contain 36 and 26 uracil residues, respectively. The dual-labeled probe used for detection of heterologous competitor RNA (and amplicon DNA derived from the competitor RNA) was: 5'-HEX-TGTGCTGCAAGGCGATTAAGTTGGGT-BHQ2-3'. All oligonucleotide primers and dual-labeled probes were synthesized by Integrated DNA Technologies, Inc. (Coralville, IA). Negative control reactions, in which RNA extracted from normal (unaffected) swine tissues or serum was added as template to the RT-PCR reaction mixture, did not produce a signal for the quantitative RT-PCR assay.

### Preparation of heterologous competitor RNA

Specific oligonucleotide primer binding sites for the PRRSV real-time RT-PCR assay were incorporated as 5' extensions in PCR primers and *in vitro *transcribed heterologous competitor RNA was prepared and spectrophotometrically quantified using methods previously described [18].

### Viral RNA extraction

Extraction of Porcine arterivirus RNA from cell culture stocks of the vaccine strain Ingelvac MLV (derived by serial passage of U.S. prototype strain VR-2332) was performed using the Qiagen viral RNA kit (Qiagen, Inc., Valencia, CA) according to the manufacturer's instructions. Viral stocks were prepared in MARC-145 cells maintained in Dulbecco's Modified Eagle medium supplemented with 10% heat-inactivated fetal bovine serum and 2 mM L-glutamine, 0.25 μg/ml fungizone, and 0.5 mg/ml gentamycin (all supplied by Mediatech, Inc., Herndon, VA). The viral-infected cell cultures were maintained at 37°C in a humidified 5% CO_2 _incubator for approximately 2 days until viral cytopathic effect was readily identified throughout the culture. Extraction of heterologous competitor RNA after in vitro transcription was performed using the Qiagen RNeasy kit (Qiagen, Inc., Valencia, CA).

### UNG treatment of reactions prior to RT-PCR

The indicated concentrations of heat-labile uracil-DNA glycosylase (Roche Applied Science, Indianapolis, IN), purified from the psychrophilic marine organism BMTU 3346 [13] were added to the amplification master mix prior to dispensing into individual amplification tubes. Samples were then held in the thermocycler at the indicated temperatures for the indicated times prior to RT-PCR. At the end of the incubation, the thermocycler was programmed to ramp the samples at the maximum rate (2.2°C/sec) to 55°C and hold at this temperature for 5 min prior to the 50°C, 30 min RT step. Control reactions, which did not contain UNG and were not subjected to incubation prior to RT-PCR, were placed in the thermocycler at the beginning of the 55°C phase, prior to the 50°C, 30 min RT step.

## Competing interests

The author(s) declare that they have no competing interests.
